# Diabetes management behaviors associated with depression in the U.S.

**DOI:** 10.1186/s13098-022-00953-3

**Published:** 2022-11-23

**Authors:** Tim C. Lai, Cassidi C. McDaniel, Chiahung Chou

**Affiliations:** 1 Department of Health Outcomes Research and Policy, Auburn University Harrison College of Pharmacy, 4306 Walker Building, Auburn, AL 36849 USA; 2 Department of Medical Research, China Medical University Hospital, Taichung City, Taiwan

**Keywords:** Diabetes mellitus, Diabetes care management, Depression, Mental distress

## Abstract

**Background:**

There is a lack of nationally representative evidence from the U.S. investigating the relationships between depression and diabetes management behaviors. Our study aimed to assess the associations between diabetes management behaviors and depression status, and to compare U.S. population-level percentages of diabetes management behaviors among patients with and without depression.

**Methods:**

A cross-sectional study was conducted using population-based survey data to assess patient-reported variables retrospectively. We used the Behavioral Risk Factor Surveillance System (BRFSS) data and included states in the U.S. that continuously adopted the diabetes optional modules in 2013, 2015, 2017, and 2019. We included U.S. adults (≥ 18 years old) with self-reported diabetes in our analysis. Main outcomes were diabetes management behaviors (i.e., self-check for blood glucose and feet sores/irritation, regular diabetes clinical visit, HbA1c check, professional feet check, and dilated eye examination) and lifestyle behaviors (i.e., exercise, smoking, and alcohol consumption).

**Results:**

Among the 74,011 respondents with diabetes, patients with depression had a higher likelihood of performing routine HbA1c checks (adjusted odds ratio (AOR) = 1.12; 95% CI 1.01–1.23) but had a lower likelihood to perform regular self-check for blood glucose (AOR = 0.91; 95% CI 0.84–0.99), receive professional feet checks (AOR = 0.87; 95% CI 0.79–0.95), and receive a dilated eye examination (AOR = 0.89; 95% CI 0.82–0.98). For lifestyle behaviors, patients with depression were more likely to smoke (No smoking (AOR) = 0.65; 95% CI = 0.59–0.72) and less likely to engage in sufficient exercise time (AOR = 0.69; 95% CI 0.63–0.75). There were no significant associations between depression and other behaviors, including self-check for feet sores/irritation (AOR = 0.99; 95% CI 0.92–1.08), regular diabetes clinical visit (AOR = 1.03, 95% CI 0.94–1.13), and alcohol consumption (AOR = 1.01, 95% CI 0.92–1.10).

**Conclusions:**

The association between depression status and diabetes management behaviors varied. People with depression were positively associated with HbA1c checks. However, less uptake of other behaviors may indicate the needs for improvement in diabetes management.

**Supplementary Information:**

The online version contains supplementary material available at 10.1186/s13098-022-00953-3.

## Introduction

The prevalence of diabetes mellitus (we used “diabetes” after) has steadily increased worldwide [1]. In the U.S., about 37.3 million people have diabetes, accounting for 11.3% of the total population [2]. Like many other chronic diseases, people with diabetes are more likely to develop mental disorders that usually increase the disease burden [3]. As “one of the most serious mental health comorbidities associated with diabetes,” [4] depression is the most well-known for its high correlation with diabetes [4–9]. However, even with such a high association, depression accompanying diabetes has often been overlooked in real-world practice [10]. When left untreated, depression could negatively influence diabetes management behaviors and diabetes control [11, 12].

To better manage diabetes, in addition to pharmacotherapy, the American Diabetes Association (ADA) suggests more comprehensive care to facilitate diabetes management behaviors, such as lifestyle changes, self-monitoring, and routine preventive visits [13]. Accordingly, the ADA provided some evidence-based criteria for each behavior as guidance for patients to follow (e.g., daily self-monitoring of blood glucose and feet sores or irritation, routine clinic visits, etc.) [13]. Uptake of diabetes management behaviors and disease control may be further complicated when depression arises and negatively affects patients’ management behaviors [4], so the ADA recommends routine mental health assessments to meet patients’ needs and optimize outcomes proactively [14, 15].

Across the U.S. population of patients with diabetes, little is known about the extent that patients utilize these diabetes management behaviors based on their mental health status (e.g., with depression vs. without depression). Previous studies examined how depression was related to diabetes management, but the study populations were mainly sampled from a few clinical settings or some regional areas [12, 16]. It is unclear how generalizable the findings from these prior studies might be to the larger diabetes population across the U.S. Since diabetes remains a significant public health concern given its high prevalence [2], population-based estimates of diabetes management behaviors by depression status will provide evidence for diabetes care and management that applies to the general adult with diabetes in the U.S.

In this study, we (1) assessed the associations between diabetes management behaviors and depression status, and (2) compared U.S. population-level percentages of diabetes management behaviors among patients with and without depression.

## Methods

### Study design and data sources

We conducted a retrospective, cross-sectional study using data from the Behavioral Risk Factor Surveillance System (BRFSS). The BRFSS is a telephone survey administered by the Population Health Surveillance Branch of the Centers for Disease Control and Prevention (CDC) [17]. The survey annually assesses health-related risk behaviors, chronic conditions, and preventive care usage from U.S. adults. The median response rate of the BRFSS survey was between 43 and 49% [18]. In this study, we only included data from states adopting the diabetes module continuously in 2013, 2015, 2017, and 2019. We chose odd years based on the higher adoption of the diabetes module across various states. We followed the Strengthening the Reporting of Observational Studies in Epidemiology (STROBE) guidelines for the reports of this study [19].

### Study population

Adult (≥ 18 years old) respondents with self-reported diabetes were included in this study based on the following survey question: “(Ever told) you had diabetes?” Respondents who answered “yes” were included, while respondents who answered any of the following were excluded: “yes, but only during pregnancy,” “don’t know,” “refused,” or missing.

### Outcome measures

The outcomes assessed included diabetes management behaviors (i.e., self-check for blood glucose and feet sores/irritation, regular diabetes clinical visit, HbA1c check, professional feet check, and dilated eye examination) and lifestyle behaviors (i.e., exercise, smoking, and alcohol consumption). The selection of these nine behaviors was based on questions from the BRFSS core and diabetes modules [20]. For the remainder of this report, we refer to these nine behaviors simply as diabetes management behaviors. Each outcome variable was recoded as a binary variable. The recommended frequencies for behaviors were as follows: self-check feet sores or irritation (≥ once per day vs. < once per day), self-glucose check (≥ once per day vs. < once per day), biannual diabetes clinical visit (≥ 2 times per year vs. < 2 times per year), biannual HbA1c check (≥ 2 times per year vs. < 2 times per year), annual feet check by a professional (≥ 1 time per year vs. < 1 time per year), annual dilated eye examination (≥ 1 time per year vs. < 1 time per year), alcohol consumption (no/moderate use vs. use), smoking (no use vs. use), and proper exercise (≥ 150 min per week vs. < 150 min per week) [13]. Please see Additional file 1: Table S1 for detailed survey questions for each outcome variable.

### Independent variables/covariates

Each outcome was assessed according to depression status (with depression vs. without depression). The depression status was based on the following question: “(Ever told) you had a depressive disorder (including depression, major depression, dysthymia, or minor depression)?” Hereinafter, our mentions of depression refer to a history of depression. Respondents who answered “don’t know” or “refused” or missing responses were taken as missing data and excluded from the study (0.5% missing; 394 out of 74,011 study population).

We assessed mental distress level using the following question: “For how many days during the past 30 days was your mental health not good?” Respondents may answer a number of poor mental health days between 0 and 30, “refuse,” “unknown,” or missing. We categorized respondents’ poor mental health days into different mental distress levels using the following criteria: no distress (0 days of poor mental health), low distress (1–13 days of poor mental health), and high distress ($\ge$14 days of poor mental health) [21]. Mental distress level was assessed because it was previously shown to be associated with depression [21, 22], and mental distress could also be a potential hinderance to perform diabetes management behaviors [23]. For mental distress, we also excluded the “refused,” “unknown,” or missing data as we did for the depression variable (2.3% missing; 1707 out of 74,011 study population).

For covariates, we included the following social-demographic characteristics: age, sex, race/ethnicity, marital status, education, geographical region of residency, employment status, and income. We also considered “received diabetes education” and “had access to care” as covariates since both variables could influence diabetes management outcomes [24, 25]. Please see Additional file 1: Table S2 for detailed survey questions for covariates.

### Missing data

#### Dependent/outcome variables

A previous study evaluated the outcome variables used by our research and found a consistent pattern for missing data, pointing out the data may be missing due to interviewers skipping the questions [26]. Hence, we excluded missing data during analyses but reported the proportion of missingness for each behavior outcome in Additional file 1: Table S1.

#### Covariates

We applied the Hot-deck imputation methods to replace missing social-demographic variables, such as age, sex, race/ethnicity, marital status, income, etc., assuming data were missing at random (MAR). Hot-deck imputation is a method replacing missing data with responses from respondents with similar characteristics [27]. The imputation was performed before selecting the study population. We also reported the proportion of missing values for each covariate in the Additional file 1: Table S2. For the “healthcare access” and “diabetes education” variables, responses of “refused,” “unknown,” or missing data were all categorized as “unknown” and remained in data analyses.

### Statistical analysis

Descriptive analyses were applied to compare demographic characteristics of those with and without depression. We evaluated the percentages of diabetes management behaviors based on respondents’ depression status and mental distress levels. We used the unadjusted and adjusted logistic regression to assess the associations between diabetes management behaviors and depression. The complex sampling design of BRFSS was incorporated during analyses, and samples were weighted to represent the state population. An a priori level of significance was set at 0.05, and hypothesis tests were 2-sided. Analyses were completed using SAS, version 9.4 (SAS Institute, Cary, NC, USA).

#### Sensitivity analysis

The missing values of covariates in the adjusted logistic regression were imputed based on the assumption that the data was MAR. However, studies showed that the assumption of MAR for income data may not hold [28, 29]. For example, the population with missing income data may be more likely to be younger, less educated, unmarried, or to have received delayed care [29]. We conducted sensitivity analyses incorporating worst-case and best-case scenarios (i.e., imputing missingness to the lowest and the highest income category), and evaluated the estimates of depression under different income assumptions [30, 31].

## Results

### Population characteristics

A total of 74,011 respondents with self-reported diabetes status were included in this study, accounting for about 10.9% (95% CI 10.7–11.1) of the survey population. Among people with diabetes, 19,508 (27.1%, 95% CI 26.4–27.9) also self-reported depression. Compared to those without depression, people with self-reported depression were more likely to be < 65 years old, female, unmarried, with a lower education level (did not graduate from high school), unemployed or with other employment status, and with a lower income level (< $20,000) (Table 1). There were no significant differences by depression status for the geographic region of residency and healthcare access. People with depression had a significantly higher percentage of receiving diabetes education compared to those without depression (54.4% vs. 51.9%; *P* = 0.01).

**Table 1:** Demographic characteristics of diabetes population, stratified by self-reported depression status

Demographic	Weighted% of DM population (SD)	DM patient without depression		DM patient with depression	
		*N*	Weighted % (95% CI)	*N*	Weighted % (95% CI)
**Total no.**	73,617^a^	54,109	–	19,508	–
Age*					
18–24	1.0 (0.08)	205	0.9 (0.7, 1.1)	118	1.5 (1.1, 1.9)
25–34	3.7 (0.23)	762	3.4 (2.8, 3.9)	494	4.5 (3.9, 5.2)
35–44	8.6 (0.29)	2203	7.9 (7.2, 8.5)	1299	10.8 (9.6, 11.9)
45–54	17.7 (0.36)	6010	15.8 (15.0, 16.6)	3433	23.0 (21.5, 24.4)
55–64	27.4 (0.39)	13,190	26.2 (25.3, 27.1)	6403	30.7 (29.2, 32.1)
65 +	41.5 (0.40)	31,739	46.0 (45.0, 46.9)	7761	29.6 (28.3, 30.9)
Sex**					
Female	49.3 (0.43)	27,787	44.6 (43.6, 45.5)	12,827	62.2 (60.6, 63.7)
Race**					
White	64.2 (0.44)	39,087	63.5 (62.5, 64.6)	14,423	66.2 (64.5, 67.8)
Black or African American	16.2 (0.33)	7304	16.6 (15.8, 17.4)	2126	15.0 (13.7, 16.3)
Asian	1.7 (0.18)	676	2.0 (1.6, 2.4)	91	0.9 (0.2, 1.7)
American Indian/Alaskan Native	2.0 (0.12)	1607	1.7 (1.5, 2.0)	673	2.6 (2.1, 3.2)
Hispanic	14.3 (0.40)	3873	14.7 (13.8, 15.7)	1477	13.1 (11.7, 14.4)
Other	1.6 (0.08)	1562	1.4 (1.3, 1.6)	718	2.2 (1.9, 2.6)
***Marital status***^b^**					
Married	58.1 (0.42)	29,031	61.2 (60.2, 62.1)	8695	49.6 (48.1, 51.2)
Single	41.9 (0.42)	25,078	38.8 (37.9, 39.8)	10,813	50.4 (48.8, 51.9)
Education**					
Did not graduate high school	20.7 (0.41)	6203	19.8 (18.9, 20.7)	2734	23.2 (21.7, 24.8)
Graduated high school	32.2 (0.38)	18,380	32.4 (31.5, 33.3)	6432	31.6 (30.2, 32.9)
Some college/tech school	30.3 (0.40)	15,052	29.6 (28.7, 30.6)	5979	31.9 (30.4, 33.3)
College/tech graduate or above	16.8 (0.27)	14,474	18.2 (17.6, 18.8)	4363	13.4 (12.4, 14.3)
***Employment***^c^**					
Unemployed	4.9 (0.22)	1740	4.2(3.7, 4.7)	1081	6.9 (6.0, 7.8)
Employed or self-employ	34.0 (0.42)	16,944	37.9 (36.9, 38.9)	4332	23.0 (22.0, 24.5)
Others	61.1 (0.43)	35,425	57.8 (56.8, 58.8)	14,095	70.1 (68.4, 71.3)
Income**					
< $20,000	26.1 (0.38)	12,458	22.9 (22.0, 23.7)	6923	34.7 (33.2, 36.2)
$20,000 to <$35,000	24.7 (0.38)	13,551	24.1 (23.2, 25.0)	4976	26.2 (24.8, 27.7)
$35,000 to <$50,000	14.2 (0.28)	8522	14.9 (14.3, 15.6)	2531	12.0 (11.2, 12.9)
$50,000 to <$75,000	13.6 (0.29)	8042	14.3 (13.7, 15.0)	2250	11.8 (10.7, 13.0)
≥$75,000	21.4 (0.35)	11,536	23.8 (22.9, 24.6)	2828	15.2 (14.1, 16.2)
***Geographic region***					
South	57.1 (0.28)	21,769	57.3 (56.6, 58.0)	7960	56.7 (55.4, 58.1)
Northeast	11.2 (0.15)	2728	11.3 (10.9, 11.7)	984	10.8 (10.0, 11.7)
Midwest	28.9 (0.21)	23,843	28.7 (28.1, 29.3)	8460	29.6 (28.5, 30.6)
West	2.6 (0.03)	4911	2.5 (2.4, 2.6)	1927	2.8 (2.6, 2.9)
U.S. Territories	0.1 (0.01)	858	0.17 (0.15, 0.18)	177	0.1 (0.08, 0.12)
***Care access***^d^					
Yes	96.57 (0.24)	53,256	96.51 (95.9, 97.1)	19,174	96.74 (96.0, 97.4)
No	3.39 (0.24)	840	3.45 (2.87, 4.03)	331	3.23 (2.52, 3.93)
Unknown	0.04 (0.02)	13	0.04 (0.00, 0.10)	3	0.03 (0.00, 0.06)
***DM education***^e^*					
Yes	52.6 (0.43)	29,872	51.9 (51.0, 53.0)	11,250	54.4 (52.8, 56.0)
No	43.2 (0.43)	22,861	44.1 (43.1, 45.1)	7754	40.9 (43.1, 45.1)
Unknown	4.2 (0.21)	1376	5.0 (3.5, 4.4)	504	4.7 (3.8, 5.7)

DM, Diabetes Mellitus; Rao-Scott Chi-Square: *P < 0.01 **P < 0.001

^a^The number excluded respondents with missing depression data. (Missingness = 394, around 0.5% of the study population)

^b^Marital status: the *Married* category included “Married,” “Separated;” the *Single* category included “Divorced,” “Widowed,” “Separated,” “Never Married,” and “A Member Unmarried Couple.”

^c^Employment: The *Others* category included “Homemaker,” “Students,” “Retired,” and “Unable to work.”

^d^*Care access* means respondents with “any kind of health insurance” or “perceived having a personal doctor or healthcare provider.”

^e^The goal set by Healthy People 2030 was 55.2%. (Ref: U.S. Department of Health and Human Services. Healthy People 2030. Accessed from: https://health.gov/healthypeople/objectives-and-data/browse-objectives/diabetes [Last accessed: 07/21/2022].)

### Unadjusted associations between diabetes management behaviors and depression

People with depression were more likely to self-check for blood glucose, self-check for feet sores or irritation, and receive regular HbA1c checks than those without depression. However, people with depression were less likely to receive feet sores or irritation checks by a professional or receive routine dilated eye examinations than those without depression. Compared to those without depression, people with depression were more likely to have no or moderate alcohol consumption, but less likely to have no smoking and sufficient exercise. (Table 2).

**Table 2:** Diabetes-related behavior among the diabetes population, comparison between people with and without depression

Behaviors	Weighted % (95% CI)	*P*-value	OR (95% CI)	*P*-value	AOR (95% CI)^a^	*P*-value
***Self-check feet sores or irritation at least 1 time/day***						
No depression	59.7 (58.7, 60.7)	0.004	Ref	–	Ref	–
Depression	62.4 (60.9, 64.0)		1.12 (1.04, 1.21)	0.004	0.99 (0.92, 1.08)	0.87
***Self-check blood glucose at least 1 time/day***						
No depression	61.3 (60.3, 62.3)	0.02	Ref	–	Ref	–
Depression	63.6 (62.0, 65.1)		1.10 (1.02, 1.19)	0.02	0.91 (0.84, 0.99)	0.03
***DM visit at least 2 times/year***						
No depression	73.5 (72.6, 74.4)	0.18	Ref	–	Ref	–
Depression	74.7 (73.3, 76.0)		1.06 (0.97, 1.16)	0.18	1.03 (0.94, 1.13)	0.53
***HbA1c check at least 2 times/year***						
No depression	71.2 (70.2, 72.2)	0.01	Ref	–	Ref	–
Depression	73.6 (72.0, 75.1)		1.13 (1.03, 1.24)	0.01	1.12 (1.01, 1.23)	0.03
***Feet sores or irritation checked by a health professional at least 1 time/year***						
No depression	76.3 (75.4, 77.2)	< 0.001	Ref	–	Ref	–
Depression	73.2 (71.7, 74.6)		0.85 (0.78, 0.93)	< 0.001	0.87 (0.79, 0.95)	0.004
***Dilated eye examination at least 1 time/year***^b^						
No depression	71.3 (70.3, 72.2)	< 0.001	Ref	–	Ref	–
Depression	65.5 (64.0, 67.0)		0.77 (0.71, 0.83)	< 0.001	0.89 (0.82, 0.98)	0.01
***No or moderate alcohol consumption***						
No depression	64.9 (63.9, 65.9)	< 0.001	Ref	–	Ref	–
Depression	70.5 (70.0, 72.0)		1.29 (1.19, 1.40)	< 0.001	1.01 (0.92, 1.10)	0.92
***No smoking***						
No depression	87.3 (86.5, 88.0)	< 0.001	Ref	–	Ref	–
Depression	75.7 (74.4, 77.1)		0.46 (0.41, 0.50)	< 0.001	0.65 (0.59, 0.72)	< 0.001
***Exercise more than 150 min/week***						
No depression	37.7 (36.7, 38.7)	< 0.001	Ref	–	Ref	–
Depression	28.1 (26.6, 29.6)		0.65 (0.59, 0.70)	< 0.001	0.69 (0.63, 0.75)	< 0.001

OR, odds ratio; AOR, adjusted odds ratio

^a^Adjusted covariates: age, sex, race, marital, education, geographical region, employment, income, diabetes education, care access

^b^The goal set by Healthy People 2030 was 70.3%. (Ref: U.S. Department of Health and Human Services. Healthy People 2030. Accessed from: https://health.gov/healthypeople/objectives-and-data/browse-objectives/diabetes [Last accessed: 07/21/2022].)

### Adjusted associations between diabetes management behaviors and depression

The adjusted logistic regression showed that people with depression had 1.12 higher odds of receiving routine HbA1c checks after adjusting for covariates. People with depression were less likely to perform regular self-check for blood glucose, receive regular feet sores/irritation checks by a professional, receive regular dilated eye examination, not smoke, and engage in sufficient exercise time. Based on the sensitivity analysis, the estimations for each outcome did not vary under different income imputation methods (Table 2).

### Unadjusted associations between diabetes management behaviors and mental distress

**Fig. 1:**
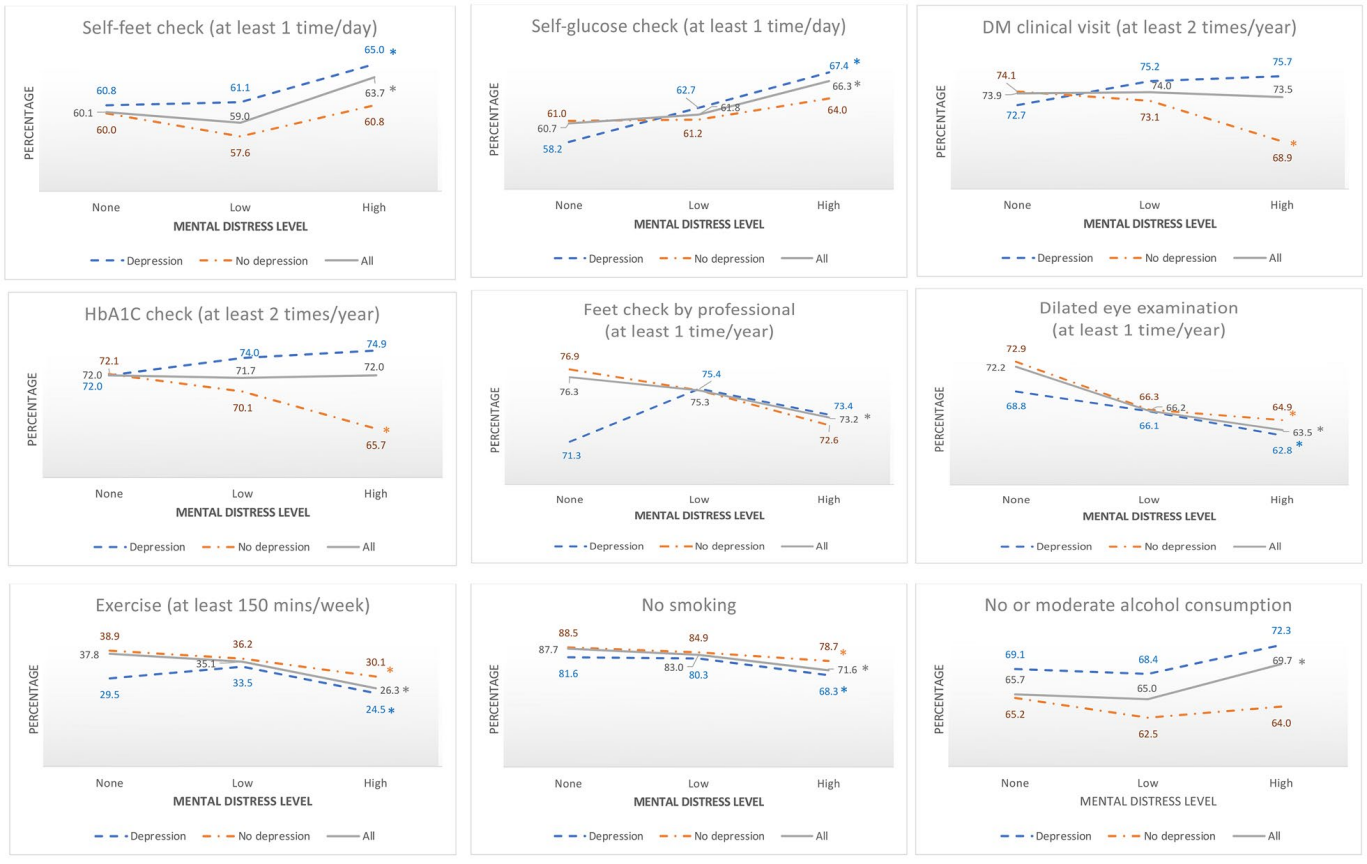
Percentage of diabetes management behaviors at different mental distress levels among populations with diabetes. *Indicates a significant difference exists between different levels of mental distress (none vs. low vs. high, *p* < 0.05). Mental distress was categorized according to the following criteria: no mental distress (0 days of poor mental health), low mental distress (1–13 days of poor mental health), and high mental distress (≥ 14 days of poor mental health) [21]

The percentages for most diabetes management behaviors were significantly different based on mental distress levels (except regular diabetes clinical visits and routine HbA1c checks); see Fig. 1. The high mental distress level showed a higher percentage of regular self-check blood glucose, routine self-check for feet sores or irritation, and no/mild alcohol consumption. However, the high mental distress level was also related to a lower percentage of having feet checked by a professional, having regular dilated eye examination, sufficient exercise, and no smoking. Finally, if only focusing on patients without depression, the percentage decreased at the high mental distress level regarding having regular diabetes clinical visits and HbA1c checks (Fig. 1).

## Discussion

The National Institute of Mental Health recognizes the higher prevalence of depression in younger age groups [32], which supports our findings of a higher depression percentage among adults < 65 years old in this nationally representative study. The association between depression status and diabetes management behaviors varied. People with depression were positively associated with HbA1c checks. Less uptake of other behaviors may indicate the needs for improvement in diabetes management.

Previous literature showed mental illness might be more influential and have a negative impact on lifestyle behaviors, such as diet, exercise, or medication adherence, that required patients’ perseverance and were more challenging to maintain [12, 33]. The conclusions from previous studies may not apply to alcohol consumption since we found no statistically significant difference between diabetes patients with and without depression. In terms of diabetes self-monitoring behaviors, studies showed mixed findings. Lin et al. concluded no significant difference between diabetes patients with and without depression regarding self-glucose checks and feet checks [12]; however, Gonzalez et al. concluded that statistical significance could be found from self-glucose checks but not from self-feet checks [16], which our study results (the adjusted associations) were congruent with. We suspected that the mixed findings regarding diabetes self-monitoring behaviors might be related to patients’ self-efficacy level, a crucial factor proven to be highly correlated with patients’ self-care behaviors [34].

Improving self-efficacy may mitigate the negative impacts of poor mental health conditions [25, 35, 36], and diabetes education has been proven to be an effective intervention to increase patients’ self-efficacy for diabetes care [35]. From Table 1, we observed that diabetes education was received more often among patients with depression (54.4%) than those without depression (51.9%). Hence, this finding could reflect a potential relationship between patients’ depression status and their self-efficacy for attending diabetes education. Also, a higher percentage of diabetes education among patients with depression might help explain why they had higher percentages for performing some diabetes management behaviors in unadjusted analyses (i.e., self-check for blood glucose, self-check for feet sores/irritation, and no/mild alcohol consumption).

Compared to patients without depression, those with depression had a lower percentage for feet checks by a professional and dilated eye examination, pointing to potential clinical practice gaps. Literature indicated that potential barriers with adherence to diabetes care might be associated with a lack of healthcare access, patient awareness or knowledge, and providers perceived lower priority than other care [37–41]. From Table 1, we knew there was not a statistically significant difference in care access between diabetes patients with and without depression. As mentioned above, patients with depression were more likely to receive diabetes education. Furthermore, the results showed that there was no statistically significant difference between diabetes patients with and without depression regarding diabetes clinical visits. Literature showed that lower priority may be given to routine professional feet checks in diabetes care [40]. Based on our findings, clinical practitioners may consider paying more attention to promoting certain diabetes management behaviors, such as routine professional feet checks and dilated eye examination.

Finally, given the high correlation between mental distress and depression provided by previous literature, we hypothesized that patients with high mental distress but without self-reported depression might potentially be patients with undiagnosed depression [10]. This hypothesis directed our research to explore different mental distress levels stratified by depression status. The figure showed that diabetes patients without depression but with high-level mental distress had a lower percentage of receiving regular diabetes clinical visits and routine HbA1c checks than those with no or low mental distress. Conversely, patients with depression had no statistically significant difference among different mental distress levels for diabetes clinical visits and regular HbA1c checks. One potential reason behind the differential trends between the two subgroups might be because care providers tended to pay more attention to monitoring patients’ mental health and provide related interventions during routine care when treating patients with mental illness history [42]. Our results indicated that among patients without depression, high mental distress was associated with nonadherence to diabetes care, which highlighted the importance of monitoring a patient’s mental health regardless of mental illness history for timely interventions.

## Limitations

This study had some limitations that need to be acknowledged. First, based on the nature of the cross-sectional study, the relationship between mental health and each diabetes management behavior could only be an association instead of a causation. In this observational study, determining causality was not possible. However, we did not intend to investigate any causality since the primary purpose of this study was to evaluate the guideline-based percentage for diabetes management behaviors. Second, the recommendation targets were general standards rather than tailoring for individualized diabetes care and management (e.g., tailoring by diabetes type, treatment regimen, or diabetes duration). The latter would be more challenging to assess due to limited clinical information provided in BRFSS. Third, since BRFSS is survey data, all variables are self-reported and susceptible to some bias. For example, disease status is not medically diagnosed, and reports of management behaviors may suffer from recall or social desirability bias. Fourth, since our samples were selected from 24 states or territories that adopted diabetes modules continuously during 2013, 2015, 2017, and 2019, the sample might not be representative of the national U.S. population. However, we found the estimated prevalence of diabetes (10.9%) from our study to be close to the national estimation (11.3%) in the U.S [2]. Lastly, medication taking was not assessed in this study because medication taking is not measured in the BRFSS data.

## Conclusion

Depression was associated with lower uptake of some diabetes management behaviors, including self-check for blood glucose, exercise, smoking, routine feet check by a professional, and dilated eye examination. Thus, more efforts could be needed to improve the uptake or receipt of these behaviors to promote optimal diabetes management among populations with depression. Findings also call for further attention to diabetes management behaviors among patients without a history of depression but with high mental distress. These patients might be more susceptible to suboptimal diabetes care and undesirable outcomes.

## Supplementary Information

**Additional file 1. Table S1.** BRFSS survey questions for the diabetes management behaviors and the number of missing values. **Table S2.** BRFSS survey questions for covariates and the number of missing values.

## Data Availability

The dataset generated and/or analyzed during the current study is available in the CDC official website, https://www.cdc.gov/brfss/index.html

## Abbreviations

AOR: Adjusted Odds Ratio
ADA: American Diabetes Association
BRFSS: Behavioral Risk Factor Surveillance System
CDC: Centers for Disease Control and Prevention
STROBE: Strengthening the Reporting of Observational Studies in Epidemiology
MAR: Missing at Random
