# Supplementary material for: Diabetes management behaviors associated with depression in the U.S
Source: Diabetol Metab Syndr. 2022 Nov 23;14:178. doi: 10.1186/s13098-022-00953-3 (PMC9685969; doi:10.1186/s13098-022-00953-3)
Supplement: Supplementary file 1 — Additional file 1. Table S1. BRFSS survey questions for the diabetes management behaviors and the number of missing values. Table S2. BRFSS survey questions for covariates and the number of missing values. [file 13098_2022_953_MOESM1_ESM.docx]

**Additional File**

**Table 1. BRFSS survey questions for the diabetes management behaviors and the number of missing values**

**Table 2. BRFSS survey questions for covariates and the number of missing values**

**Table 1**. **BRFSS Survey questions for the diabetes management behaviors and the number of missing values**

| **Variable** | **Survey Question(s)^a^** | **Missing data** | **Missing %*** |
| --- | --- | --- | --- |
| Self-check blood glucose | About how often do you check your blood for glucose or sugar? | 3026 | 4.11% |
| Self-check foot sores/irritations | Including times when checked by a family member or friend, about how often do you check your feet for any sores or irritations? | 4377 | 5.95% |
| DM clinical visits | About how many times in the past 12 months have you seen a doctor, nurse, or other health professional for your diabetes? | 3975 | 5.40% |
| A1C check | About how many times in the past 12 months has a doctor, nurse, or other health professional checked you for A-one-C? | 5977 | 8.12% |
| Feet check by a health professional | About how many times in the past 12 months has a health professional checked your feet for any sores or irritations? | 4229 | 5.74% |
| Dilated eye examination | When was the last time you had an eye exam in which the pupils were dilated, making you temporarily sensitive to bright light? | 2810 | 3.82% |
| Smoking | 1. Have you smoked at least 100 cigarettes in your entire life?  2. Do you **NOW** smoke cigarettes every day, some days, or not at all? (*If respondents answered “Not at all”, we also categorized them as non-smokers*) | 2473 | 3.36% |
| Alcohol consumption | During the past 30 days, how many days per week or per month did you have at least one drink of any alcoholic beverage such as beer, wine, a malt beverage or liquor? | 3101 | 4.21% |
| Exercise | 1. During the past month, other than your regular job, did you participate in any physical activities or exercises such as running, calisthenics, golf, gardening, or walking for exercise?  2. How many times per week or per month did you take part in this activity during the past month?  3. And when you took part in this activity, for how many minutes or hours did you usually keep at it? | 4592 | 6.24% |
| *Sample size = 73,617 | | | |

^a^ Reference: Centers for Disease Control and Prevention (CDC). *Behavioral Risk Factor Surveillance System Survey Questionnaire*. Atlanta, Georgia: U.S. Department of Health and Human Services, Centers for Disease Control and Prevention. *Accessed June, 2022*.

**Table 2. BRFSS survey questions for covariates and the number of missing values**

| **Variable** | **Survey Question(s)^a^** | **Missing data** | **Missing %*** | **Note** |
| --- | --- | --- | --- | --- |
| Age | See Note | 0 | 0.00% | We used the imputed age variable provided by the BRFSS dataset. |
| Sex | What is your sex? or What was your sex at birth? Was it...(Multiple choice) | 63 | 0.01% |  |
| Geographic location | See Note | 0 | 0.00% | We categorized to Census regions based on respondents’ state of residence |
| Race | See Note | 2021 | 0.36% | We used the imputed race variable provided by the BRFSS dataset in the years 2013, 2017, and 2019; however, BRFSS didn’t provide imputed race information in 2015. |
| Education level | What is the highest grade or year of school you completed? (Multiple choice) | 2065 | 0.37% |  |
| Employment status | Are you currently...? (Multiple choice) | 5105 | 0.91% |  |
| Marital status | Are you: (Multiple choice) | 3840 | 0.68% |  |
| Annual income | Is your annual household income from all sources: (If respondent refuses at any income level, code “Refused”.) | 95660 | 17.05% |  |
| Care access | 1. Do you have any kind of health care coverage, including health insurance, prepaid plans such as HMOs, or government plans such as Medicare, or Indian Health Service?  2. Do you have one person you think of as your personal doctor or health care provider? (If “No” ask “Is there more than one or is there no person who you think of as your personal doctor or health care provider?”.) | 16 | 0.00% | Having care access means respondents with either having health insurance coverage” or having a personal doctor or health care provider |
| Diabetes Education | Have you ever taken a course or class in how to manage your diabetes yourself? | 1880 | 0.34% |  |
| *Sample size = 560,992 | | | | |

^a^ Reference: Centers for Disease Control and Prevention (CDC). *Behavioral Risk Factor Surveillance System Survey Questionnaire*. Atlanta, Georgia: U.S. Department of Health and Human Services, Centers for Disease Control and Prevention. *Accessed June, 2022*.
